# Comparative Effectiveness of Tibolone and Combined Hormone Therapy in Relieving Menopausal Insomnia

**DOI:** 10.1177/26884844251387909

**Published:** 2025-10-20

**Authors:** Zicheng Song, Changbin Li, Feng Jin, Dongmei Sun, Minfang Tao

**Affiliations:** ^1^ Department of Obstetrics and Gynecology, Shanghai Jiao Tong University School of Medicine Affiliated Renji Hospital, Shanghai, China.; ^2^ Department of Obstetrics and Gynecology, Shanghai Sixth People’s Hospital Affiliated to Shanghai Jiao Tong University School of Medicine, Shanghai, China.

**Keywords:** sleep disorder, menopause, PSQI, tibolone, combined hormone therapy

## Abstract

**Objective:**

This study explores the effectiveness of tibolone and combined hormone therapy (HT) in alleviating sleep disorders in women aged 40–65 who are in menopausal transition and postmenopause.

**Methods:**

The ambidirectional cohort study (retrospective 2011–2015; prospective 2016–2021), conducted at Shanghai Sixth People’s Hospital’s specialized menopause clinic. Participants were given tibolone or combined HT based on their STRAW + 10 stratification and preference for continued menstruation. Demographic information, baseline, and reevaluated performance of the Pittsburgh Sleep Quality Index (PSQI) were collected for analysis of their differentiations and impact factors. Sleep quality was reassessed 12 months after treatment initiation *via* PSQI.

**Results:**

The cohort study involved 285 participants—184 in the tibolone cohort and 101 in the combination HT cohort. After 12 months, PSQI scores improved by −3.76 ± 4.12 with tibolone and –3.66 ± 4.23 with combined HT. The adjusted between group difference was –0.359 (95% confidence interval = 0.577; *p* = 0.534).

**Conclusions:**

The study suggests that tibolone and combined HT improved sleep to a similar extent after adjustment. Further comprehensive research is necessary to corroborate these observations.

## Introduction

Menopausal symptoms can be highly disruptive for women during the transition period, which typically spans approximately 4 years before the final menstrual period.^1^ The Study of Women’s Health Across the Nation divides the Stages of Reproductive Aging Workshop (STRAW + 10) broadly into three categories, namely, reproductive, menopausal transition, and postmenopause in order to guide the diagnosis and management of menopausal symptoms.^2^ Sleep disturbances affect 40%–60% of menopausal women compared to 10.5% for hot flashes in Chinese women.^3^ Statistically, sleep disturbances are present in 40%–60% women with menopausal symptoms, rendering it one of the most irritating symptoms.^4^

Menopausal symptoms primarily result from declining estrogen production, disrupting hypothalamic pathways. Elevated follicle-stimulating hormone levels principally reflect ovarian aging rather than directly causing psychological symptoms.^5^ Randomized trials and meta-analyses show that menopausal hormone therapy (MHT) remains the most effective treatment for vasomotor symptoms and confers ancillary benefits for bone, sleep, and urogenital health.^6–8^ Previous high-quality randomized controlled trial (RCT) and meta-analysis, such as National Institute for Health and Care Excellence, The North American Menopause Society, American College of Obstetricians and Gynecologists, have demonstrated the effectiveness of MHT on attenuating menopausal symptoms, yet head-to-head data comparing individual regimens are limited—an evidence gap addressed by the present study.^9–11^

While MHT reliably relieves vasomotor symptoms,^12^ its sleep effects show scenario-dependent efficacy, with benefits mainly seen in women reporting hot flashes.^13^ The 2016 Cochrane review systematically compared tibolone and combined hormone therapy (HT), demonstrating similar but slightly reduced efficacy in alleviating vasomotor symptoms (odds ratio [OR] = 1.57, 95% confidence interval [CI]: 1.18–2.10) after adjusting for attrition bias.^14^ However, in contrast to what is hypothesized that sleeping disorders should have been significantly improved after vasomotor symptoms being controlled by MHT, Geiger’s RCT on transdermal estrogen plus progesterone for its palliation on sleep disorders found that only sleep latency (estimate = −0.12, *p* = 0.002) and sleep disturbance (estimate = −0.24, *p* = 0.04) had been demonstrated signs of improvements in comparison of placebo groups, and these effects persisted after adjustment for vasomotor intensity, implying a mechanism beyond hot-flush relief.^15^ In addition, previous studies on the effect of MHT on sleep disorders have several limitations that hinder the credibility of the MHT, including but not limited to heterogeneity in trial population, the differences of pharmacokinetics and drug metabolisms on individuals, MHT formulation variations, and so on.^12^ To address this gap, we compared tibolone (2.5 mg daily) with combined HT (oral estradiol + dydrogesterone) in peri- and postmenopausal women followed for 12 months. We hypothesized that tibolone would produce improvements in global sleep quality equivalent to those achieved with combined MHT.

## Materials and Methods

### Study participants

The study, spanning from October 2010 to July 31, 2021, at Shanghai Sixth People’s Hospital’s specialized menopause clinic, executed an ambidirectional (retrospective–prospective) cohort study involving menopausal patients: routine-care records from October 1, 2010, to May 31, 2016, were abstracted retrospectively, and eligible women seen from June 1, 2016, to July 31, 2021, were enrolled and followed prospectively. Participants were chosen through convenience sampling and included women aged 40–65 years in the peri- or postmenopausal stages as defined by STRAW + 10 criteria, without contraindications to MHT, and expressing willingness for participation. Exclusions were made for primary sleep disorders, including restless legs syndrome, obstructive sleep apnea, and narcolepsy, and for those who underwent estrogen therapy within the previous 6 months. During the study period, 1091 women attended the clinic; 766 met eligibility criteria. The Institutional Review Board (IRB) granted a waiver of consent for retrospective chart review through May 31, 2016; from June 1, 2016, onward, all participants provided written informed consent. In total, 285 women were enrolled and included in the analysis. Ethical approval for this study was granted by the Ethics Committee of Shanghai Jiao Tong University’s Sixth People’s Hospital (2016-R07; Registration Number: ChiCTR-IPR-16008754).

### Study design

This ambidirectional (retrospective–prospective) cohort study, conducted at Shanghai Sixth People’s Hospital’s specialized menopause clinic, divided participants into two treatment groups based on their STRAW + 10 stratification and preference for continued menstruation. The tibolone group received a dosage of 2.5 mg/day in the morning, while dydrogesterone 10 mg was administered once daily on days 15–28 of each 28-day cycle, with continuous oral 17β-estradiol 2 mg in the combined HT group. Both regimens were standardized for all women in this group throughout the 12-month follow-up. No participant used an alternative schedule. For participants in stages −2 and −1, combined HT was recommended. Those in stage 0 or +1 were given the choice; if they preferred to continue menstruating, combined HT was administered, otherwise, tibolone was prescribed. Participants in stages +1b, +1c, and +2 were advised to undergo tibolone therapy. This approach ensured that treatment was tailored to the individual needs and stages of each participant.

### Data collection

Demographic information including name, age, height, weight, marital status, occupation, educational level, monthly income, contraceptive history, smoking and alcohol intake history, and previous estrogen treatments was collected. Sleep disorders were assessed using the Pittsburgh Sleep Quality Index (PSQI), a standardized questionnaire administered by trained researchers, conducted twice: once before treatment initiation and again at the 1-year follow-up.

Parameters such as subjective sleep quality, sleep latency, sleep duration, habitual sleep efficiency, sleep disturbance, use of sleeping medication, daytime dysfunction, and global PSQI score were separately evaluated based on the participant’s responses to the PSQI questionnaire, which typically asks about sleep habits over the previous months. The PSQI assesses sleep quality across seven components: subjective sleep quality (overall perception of sleep, rated 0–3), sleep latency (time to fall asleep, scaled 0–3), sleep duration (hours of actual sleep, rated 0–3), habitual sleep efficiency (percentage of time spent asleep while in bed, rated 0–3), sleep disturbance (frequency of disturbances like waking up at night, rated 0–3), use of sleeping medication (frequency of medication use, scaled 0–3), and daytime dysfunction (impact of sleep on daily activities, rated 0–3). The Global PSQI Score, ranging from 0 to 21, is a summation of these components, with higher scores indicating poorer sleep quality. Overall score surpassing 5 was considered as the cutoff value to indicate sleep disorders. Patients were evaluated at baseline and posttreatment evaluation occurred at 12 months to maximize drug effect observation.

### Statistical method

The data processing involved preprocessing, statistical description, and both one-way and multivariate analyses, conducted using R (version 4.3.0). The measurement data conformed to a normal distribution and were expressed using the mean and standard deviation (SD). In cases where the data did not follow a normal distribution, the median, maximum, and minimum values were used instead. The statistical analysis included two-sample *t*-tests and analysis of variance for datasets that followed a normal distribution, and nonparametric tests were utilized for data that did not meet the assumptions of normality. The categorical data were presented as frequency and percentage, and χ^2^ tests were employed for the differences between categorical groups, as well as demographic variables, relevant treatment history, baseline PSQI scores, and changes in PSQI levels after treatment. A linear regression model was used to examine the impact of sleep alleviation of two types of drugs after adjusting for covariates that were the baseline demographic variables that differed between treatment groups. A *p*-value of <0.05 was considered statistically significant.

## Results

### Participants’ description

A total of 285 participants were recruited for this trial, 184 in the tibolone group and 101 in the combined HT group. The mean age of the participants was 53.2 years (SD = 3.8) in the tibolone group and 47.9 years (SD = 4.6) in the combined HT group. The difference in age between the two groups was statistically significant (*p* < 0.001). The mean body mass index (BMI) in the tibolone and combined HT groups was 22.15 (SD = 3.29) and 21.48 (SD = 2.41), respectively, and the difference was not significant at the 0.05 level (*p* = 0.059). Most participants (97% or more) in both groups were married. Participants in the combined HT group were more likely to be less educated and unemployed with lower income than their counterparts in the tibolone group (*p* < 0.05). Contraceptive use, smoking habits, and alcohol consumption were largely similar between the two groups. See Table 1.

**Table 1.** Basic Characteristics

Characteristic	Tibolone, *N* = 184^a^	Combined HT, *N* = 101^a^	*p*-Value^b^
Marital status			0.191
Married	182 (98.9%)	98 (97.0%)	
Single	1 (0.5%)	0 (0.0%)	
Divorce	1 (0.5%)	3 (3.0%)	
Occupation			<0.001**
Unemployed	7 (3.8%)	4 (4.0%)	
Employed	97 (52.7%)	80 (79.2%)	
Retired	80 (43.5%)	16 (15.8%)	
None	0 (0.0%)	1 (1.0%)	
Education level			0.036*
Graduate	10 (5.4%)	4 (4.0%)	
Undergraduate	78 (42.4%)	58 (57.4)	
High school	55 (29.9%)	16 (15.8%)	
Primary and secondary	41 (22.3%)	23 (22.8%)	
Monthly income			0.002**
Over 10K RMB	23 (12.5%)	20 (19.8%)	
5–10K RMB	46 (25.0%)	32 (31.7%)	
3–5K RMB	56 (30.4%)	23 (22.8%)	
1–3K RMB	52 (28.3%)	14 (13.9%)	
<1K RMB	7 (3.8%)	12 (11.9%)	
Contraception			0.139
IUD	38 (20.6%)	21 (20.8%)	
None	140 (76.1%)	74 (73.3%)	
Oral contraception	0 (0.0%)	2 (2.0%)	
Condom	0 (0.0%)	2 (2.0%)	
Oral contraception + condom	5 (2.7%)	2 (2.0%)	
Ligation	1 (0.5%)	0 (0.0%)	
History of smoking	0 (0.0%)	1 (1.0%)	0.354
History of alcohol	0 (0.0%)	1 (1.0%)	0.354

*p < 0.05; **p < 0.01.

^a^ Mean (SD); *n* (%).

^b^ Welch’s two-sample *t*-test; Fisher’s exact test; Pearson’s chi-squared test; Wilcoxon rank-sum test.

HT, hormone therapy; SD, standard deviation; IUD, intrauterine device.

### Comparison of pre- and posttreatment changes in sleep parameters

**FIG. 1.**
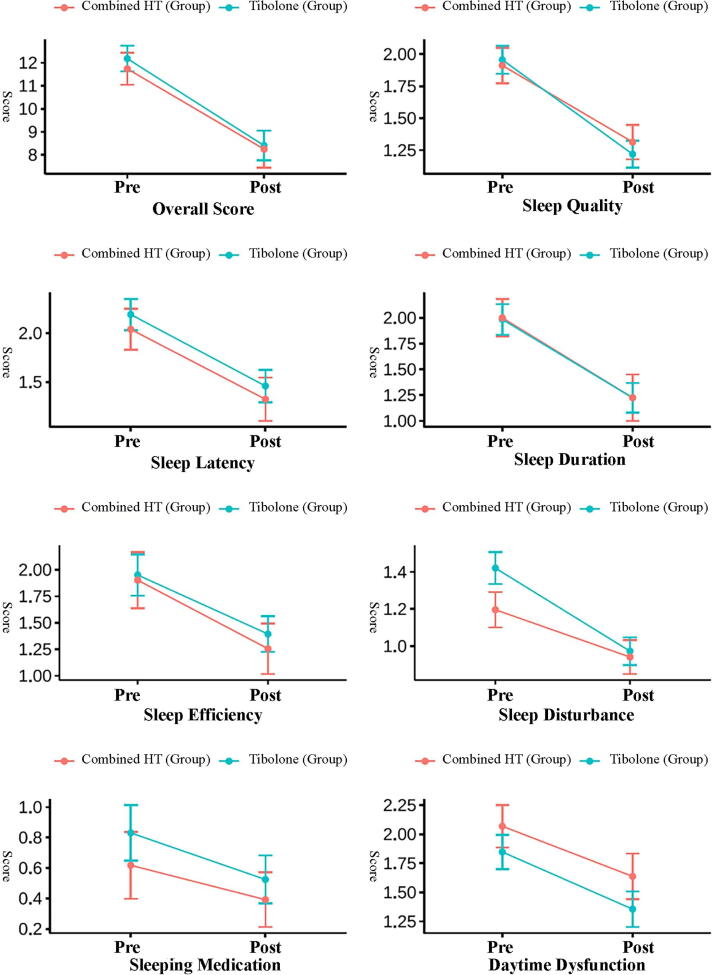
Difference in PSQI score pre- and posttreatment by treatment groups. PSQI, Pittsburgh Sleep Quality Index.

The results presented in Figure 1 are based on univariate analysis of differences in PSQI scores before and after treatment. At the 12-month follow-up visit, PSQI scores improved in both the combined HT and tibolone groups in all sleep parameter scores posttreatment (*p* < 0.001). Women in the tibolone group experienced a greater reduction in sleep disturbance scores compared to the combined HT group (mean difference: 0.45 vs. 0.26, *p* = 0.012). However, differences in other PSQI components between the two groups were not statistically significant (*p* > 0.05). Further multivariate analysis is required to confirm these findings and adjust for potential confounders.

### Univariate analysis of differences in overall scores between pretreatment and posttreatment

The univariate analysis assessed the association between potential influencing factors and the differences in overall scores (Δd) between pretreatment and posttreatment. This analysis did not identify statistically significant relationships between Δd and variables such as marital status, occupational status, education level, monthly income, contraception method, age, BMI, smoking history, or alcohol intake (all *p* > 0.05). Although some trends were observed—for instance, a positive association with oral contraception use (β = 2.941, *p* = 0.335) and a potential negative association with “Unemployed” occupational status (β = −0.905, *p* = 0.493)—none reached statistical significance. The results, based on the Δd as the dependent variable, are detailed in Table 2.

**Table 2.** Comparison of Differences in Overall Scores Between Pretreatment and Posttreatment: Results of Univariate Analysis

Characteristic	Beta	95% CI	SE	*t*	*p*-Value
Marital status					
Married					
Single	−1.311	−9.625, 7.004	4.242	−0.309	0.758
Divorce	2.189	−1.990, 6.369	2.132	1.027	0.305
Occupational status					
Unemployed					
Employed	−0.905	−3.491, 1.680	1.319	−0.686	0.493
Retired	−1.085	−3.734, 1.563	1.351	−0.803	0.423
None	−2.273	−10.963, 6.418	4.434	−0.513	0.609
Education level					
Graduate					
Undergraduate	1.011	−1.319, 3.340	1.188	0.850	0.396
High school	1.474	−0.953, 3.900	1.238	1.190	0.235
Primary and secondary	0.580	−1.868, 3.029	1.249	0.465	0.643
Monthly income					
Over 10K RMB					
5–10K RMB	0.040	−1.533, 1.613	0.803	0.050	0.960
3–5K RMB	0.891	−0.679, 2.460	0.801	1.113	0.267
1–3K RMB	1.278	−0.345, 2.901	0.828	1.543	0.124
<1K RMB	0.233	−2.049, 2.514	1.164	0.200	0.842
Contraception					
IUD					
None	−0.372	−1.593, 0.848	0.623	−0.598	0.550
Oral contraception	2.941	−3.027, 8.908	3.045	0.966	0.335
Condom	−2.059	−8.027, 3.908	3.045	−0.676	0.499
Oral contraception + condom	2.155	−1.163, 5.473	1.693	1.273	0.204
Ligation	−0.559	−8.930, 7.811	4.271	−0.131	0.896
Age	−0.005	−0.107, 0.096	0.052	−0.100	0.920
BMI	−0.016	−0.170, 0.137	0.078	−0.207	0.836
History of smoke					
No	—	—			
Yes	−1.342	−9.657, 6.974	4.243	−0.316	0.752
History of alcohol intake					
No	—	—			
Yes	−1.342	−9.657, 6.974	4.243	−0.316	0.752

BMI, body mass index; CI, confidence interval; SE, standard error; IUD, intrauterine device.

Both tibolone and combined HT produced significant improvements across all PSQI domains after 12 months of treatment (Table 3). Table 4 compares sleep-related outcomes pre- and postintervention across the overall sample (*n* = 285), tibolone group (*n* = 184), and combined HT group (*n* = 101). Sleep disturbance pre-intervention significantly differed between the groups (*p* < 0.001), with higher scores in the tibolone group. Postintervention, daytime dysfunction showed a significant difference between groups (*p* = 0.024), with the combined HT group reporting higher scores. Change scores (post–pre) indicate an overall reduction in sleep-related issues across both groups, though only changes in sleep disturbance (*p* = 0.012) reached statistical significance between groups. No significant differences were observed for the change in overall sleep scores (*p* = 0.599) or other subdomains such as sleep quality, latency, efficiency, or the use of sleep medication. These results suggest that both interventions improved sleep outcomes, but specific aspects such as sleep disturbance may differ in their impact between the treatments. See Table 4.

**Table 3.** Comparison of the Effectiveness of Sleep Alleviation Within the Tibolone and Combined Hormone Therapy Groups

Variables	Pre	Post	Statistics	*p*
Tibolone				
Overall score	12.19 ± 3.79	8.43 ± 4.40	12.376	<0.001**
Sleep quality	1.95 ± 0.75	1.22 ± 0.72	10.618	<0.001**
Sleep latency	2.19 ± 1.09	1.47 ± 1.14	7.724	<0.001**
Sleep duration	1.99 ± 1.02	1.23 ± 0.99	8.732	<0.001**
Sleep efficiency	1.96 ± 1.33	1.40 ± 1.16	4.363	<0.001**
Sleep disturbance	1.42 ± 0.60	0.97 ± 0.52	9.651	<0.001**
Use of sleeping medication	0.84 ± 1.25	0.52 ± 1.08	4.291	<0.001**
Daytime dysfunction	1.84 ± 1.01	1.35 ± 1.05	6.107	<0.001**
Combined HT				
Overall score	11.70 ± 3.57	8.22 ± 4.11	7.905	<0.001**
Sleep quality	1.90 ± 0.70	1.30 ± 0.67	7.462	<0.001**
Sleep latency	2.03 ± 1.07	1.33 ± 1.13	6.034	<0.001**
Sleep duration	1.99 ± 0.93	1.21 ± 1.13	6.351	<0.001**
Sleep efficiency	1.89 ± 1.36	1.25 ± 1.21	3.765	<0.001**
Sleep disturbance	1.20 ± 0.49	0.94 ± 0.47	4.236	<0.001**
Use of sleeping medication	0.62 ± 1.12	0.40 ± 0.92	2.184	0.031*
Daytime dysfunction	2.07 ± 0.93	1.64 ± 1.01	3.557	<0.001**

*p < 0.05; **p < 0.01.

**Table 4.** Comparison of the Effectiveness of Sleep Alleviation Between the Tibolone and Hormone Therapy Groups

Variables	Overall (*n* = 285)	Tibolone (*n* = 184)	Combined HT (*n* = 101)	Statistics	*p*
Pre					
Overall score_pre	12.02 ± 3.72	12.19 ± 3.79	11.70 ± 3.57	1.059	0.291
Sleep quality_pre	1.93 ± 0.73	1.95 ± 0.75	1.90 ± 0.70	0.553	0.581
Sleep latency_pre	2.13 ± 1.09	2.19 ± 1.09	2.03 ± 1.07	1.194	0.233
Sleep duration_pre	1.99 ± 0.99	1.99 ± 1.02	1.99 ± 0.93	0.008	0.994
Sleep efficiency_pre	1.93 ± 1.34	1.96 ± 1.33	1.89 ± 1.36	0.394	0.694
Sleep disturbance_pre	1.34 ± 0.57	1.42 ± 0.60	1.20 ± 0.49	3.441	<0.001
Use of sleeping medication_pre	0.76 ± 1.21	0.84 ± 1.25	0.62 ± 1.12	1.472	0.142
Daytime dysfunction_pre	1.92 ± 0.99	1.84 ± 1.01	2.07 ± 0.93	1.866	0.063
Post					
Overall score_post	8.35 ± 4.29	8.43 ± 4.40	8.22 ± 4.11	0.397	0.691
Sleep quality_post	1.25 ± 0.71	1.22 ± 0.72	1.30 ± 0.67	0.849	0.397
Sleep latency_post	1.42 ± 1.14	1.47 ± 1.14	1.33 ± 1.13	0.999	0.319
Sleep duration_post	1.22 ± 1.04	1.23 ± 0.99	1.21 ± 1.13	0.192	0.848
Sleep efficiency_post	1.35 ± 1.18	1.40 ± 1.16	1.25 ± 1.21	1.057	0.291
Sleep disturbance_post	0.96 ± 0.50	0.97 ± 0.52	0.94 ± 0.47	0.521	0.602
Use of sleeping medication_post	0.48 ± 1.02	0.52 ± 1.08	0.40 ± 0.92	1.039	0.300
Daytime dysfunction_post	1.46 ± 1.04	1.35 ± 1.05	1.64 ± 1.01	2.272	0.024
Differences					
Overall score (post–pre)	−3.66 ± 4.23	−3.76 ± 4.12	−3.49 ± 4.43	0.526	0.599
Sleep quality (post–pre)	−0.68 ± 0.89	−0.73 ± 0.93	−0.60 ± 0.81	1.127	0.261
Sleep latency (post–pre)	−0.72 ± 1.23	−0.72 ± 1.27	−0.70 ± 1.17	0.130	0.897
Sleep duration (post–pre)	−0.76 ± 1.19	−0.76 ± 1.17	−0.78 ± 1.24	0.180	0.857
Sleep efficiency (post–pre)	−0.59 ± 1.72	−0.55 ± 1.72	−0.64 ± 1.72	0.419	0.676
Sleep disturbance (post–pre)	−0.38 ± 0.63	−0.45 ± 0.63	−0.26 ± 0.61	2.526	0.012
Use of sleeping medication (post–pre)	−0.28 ± 1.01	−0.32 ± 1.00	−0.23 ± 1.05	0.696	0.487
Daytime dysfunction (post–pre)	−0.47 ± 1.13	−0.49 ± 1.09	−0.43 ± 1.20	0.453	0.651

### Comparison of the effectiveness between the tibolone and HT groups: Results of multivariate linear regression analyses

The data revealed the indistinct sleep alleviation effects in terms of PSQI scores differences between the tibolone group and the combined HT group. After adjustment for age, occupation, education, and income, between-group differences became nonsignificant. The total score, sleep quality, latency, duration, efficiency, use of sleeping medication, and daytime dysfunction showed no significant differences between the groups as well (*p* > 0.05). See Table 5.

**Table 5.** Comparison of the Effect of Sleep Alleviation Between the Two Drug Groups: Results of Multivariate Linear Regression Analyses^a^

	Tibolone vs. combined HT		*p*-Value
	Score difference	95% CI	
Overall score (post–pre)	−0.359	0.577	0.534
Sleep quality	−0.207	0.117	0.079
Sleep latency	−0.021	0.170	0.902
Sleep duration	−0.064	0.162	0.696
Sleep efficiency	0.092	0.236	0.697
Sleep disturbance	−0.165	0.086	0.057
Sleeping medication	−0.045	0.141	0.749
Daytime dysfunction	−0.050	0.155	0.746

^a^ Combined HT and tibolone groups were controlled in the above linear regression analyses for age, occupation, education, and income.

## Discussion

Both tibolone and cyclical estradiol plus dydrogesterone produced clinically meaningful improvements in global PSQI and in every component score. In univariate analyses tibolone yielded a slightly larger reduction in the “sleep disturbance” component than combined HT (−0.45 ± 0.63 vs. −0.26 ± 0.61, *p* = 0.012); however, after adjustment for age, STRAW stage, and socioeconomic covariates, this difference was no longer significant (β = −0.165, 95% CI = 0.086, *p* = 0.057). The convergence of posttreatment scores indicates that the baseline disturbance—rather than an intrinsic pharmacological superiority—accounts for the unadjusted change.

Tibolone was taken once daily in the morning, consistent with its mild androgenic profile, whereas 17β-estradiol was continuous and dydrogesterone was periodical (days 15–28 of each 28-day cycle). Although androgen receptor activation has been hypothesized to stabilize arousal thresholds,^16^ current evidence does not demonstrate a distinct sleep pathway for tibolone beyond relief of vasomotor symptoms. Accordingly, our data do not support preferential use of tibolone solely for insomnia when combined HT is otherwise suitable.

The sleep quality during menopause is also predominantly influenced by other endocrine and chronobiological factors.^17^ Recent studies associate elevated 24-hour urinary free cortisol,^18^ declining nocturnal melatonin,^19,20^ and age-related phase shifts in core body temperature^21,22^ with poorer sleep quality in menopausal women. Unfortunately, we could not test these pathways due to the lack of biomarker data. However, their potential contribution underscores the need for future trials that integrate objective sleep metrics with serial measures of cortisol, melatonin, and vasomotor intensity. Such multimodal designs would clarify whether adjunct circadian-based interventions—light therapy, timed exercise, or melatonin agonists—can augment the sleep benefit we observed with tibolone and combined MHT.

This study offers a rare, head-to-head comparison of tibolone and cyclical estradiol plus dydrogesterone, delivered in protocol-standardized doses and evaluated with the PSQI at baseline and 12 months. The ambidirectional design combines a decade of research data with uniform outcome measurement, with over 200 participants. The analysis demonstrates that both regimens comparably improve menopausal sleep, providing credible evidence to inform guideline-recommended, patient-centered therapy.

This study has several limitations that warrant consideration. First, treatment allocation was non-randomized and preference-based, raising the possibility of selection bias. Second, key manifestations such as hot-flush severity and mood status (*e.g.*, GAD-7, PHQ-9) were not recorded for the entire ambidirectional cohort because unmeasured vasomotor changes would likely bias toward the null, given similar efficacy between regimens,^12^ yet its absence remains a constraint. Third, recruitment from a single specialized menopause clinic restricts ethnic and socioeconomic diversity and therefore limits external validity. Fourth, the 12-month follow-up may be insufficient to characterize longer-term benefits and risks of either regimen. Last, sleep was assessed exclusively with the PSQI, a subjective instrument susceptible to recall bias.^23^ Future investigations should combine patient-reported measures with objective methodologies such as actigraphy or polysomnography. Collectively, these constraints highlight the need for multicenter, randomized trials with extended follow-up to delineate the comparative effectiveness of tibolone and combined HT as well as other MHT.

## Conclusions

The study indicates that tibolone and combined HT are equally effective in improving sleep quality after adjustment for baseline differences. Further researches are warranted to substantiate these findings.

## Abbreviations Used

χ²: chi-square
Δd: change score
ACOG: American College of Obstetricians and Gynecologists
ANOVA: analysis of variance
BMI: body mass index
CI: confidence interval
FMP: final menstrual period
FSH: follicle-stimulating hormone
GAD-7: Generalized Anxiety Disorder-7 questionnaire
HT: combined hormone therapy
IRB: Institutional Review Board
IUD: intrauterine device
MHT: menopausal hormone therapy
NAMS: The North American Menopause Society
NICE: National Institute for Health and Care Excellence
OR: odds ratio
PHQ-9: Patient Health Questionnaire-9
PSQI: Pittsburgh Sleep Quality Index
RCT: randomized controlled trial
SD: standard deviation
SE: standard error
STRAW+10: Stages of Reproductive Aging Workshop +10
SWAN: Study of Women’s Health Across the Nation
